# Detailed biogeographical mapping as a useful novel tool for the conservation of endemic taxa: a case of study for Iberian orchids

**DOI:** 10.12688/openreseurope.17737.2

**Published:** 2025-01-30

**Authors:** Angel Penas, Raquel Alonso-Redondo, Alejandro González-Pérez, Aitor Álvarez-Santacoloma, Norma Yolanda Ochoa-Ramos, Giovanni-Breogán Ferreiro-Lera, Sara del Río

**Affiliations:** 1 Biodiversity and Environmental Management (Botany Area), Universidad de Leon, León, Castile and León, 24071, Spain; 2 Mountain Livestock Institute, CSIC General Foundation, León, Castile and León, 24071, Spain

**Keywords:** Endemisms; Bioclimatology; Biogeography, Cartography, Flora Conservation, Iberian endemisms, Orchidaceae, Phytosociology, Species distribution.

## Abstract

**Background:**

Many Iberian orchids occur in plant communities designated as habitats of interest in the European Union, particularly in Mediterranean grasslands and forests. Their ecological importance highlights the need for a deeper understanding of their distribution and ecological requirements in order to develop effective conservation and management policies.

**Methods:**

This study focuses on the biogeographical mapping and characterisation of five Iberian and Balearic endemic orchid species at district level. Distribution data were collected from existing biodiversity databases and integrated into a Geographic Information System (GIS). In order to assign the correct biogeographical epithet to each taxon, a set of rules and criteria was developed to ensure an objective, simple and universal classification based on the species' distribution areas.

**Results:**

For each orchid species, the study identified its phytosociological assignment, bioclimatic range and current conservation status. Detailed maps were produced, providing insights into the biogeographical, bioclimatic and phytosociological attributes of these taxa.

**Conclusions:**

The biogeographical mapping and ecological classification presented in this study provide a basis for effective decision-making regarding the conservation and management of these orchid species. In addition, the results can be used to update their conservation status to better reflect their ecological needs and threats.

## Plain Language Summary

We looked at some specific types of orchids that grow in certain parts of Spanish Peninsula and the Balearic Islands. These orchids are important because they live in habitats that the European Union cares about, especially in the forests around the Mediterranean. We wanted to understand more about where these orchids live and what they need to survive, so that we can better protect them.

To do this, we used information from different databases about where these orchids have been found. Then we put all this information together using a special computer program called a Geographic Information System (GIS). This helped us make maps showing where these orchids are found in different areas.

We also came up with some rules and criteria to help us understand more about each type of orchid and where it lives. This includes things like what kind of climate it likes and what other plants it hangs out with. Understanding all this helps us make better decisions about how to look after these orchids and make sure they continue to grow in the future.

## Introduction

The Iberian Peninsula, located in the western Mediterranean basin, is known as a hotspot of plant biodiversity ^1– 3^. With an estimated 6,500 species and subspecies of vascular plants, including those found in the Balearic Islands, and a remarkable endemicity rate of 28% ^4, 5^, its ecological importance cannot be underestimated. However, this region is currently facing significant challenges due to global warming, which has led to an increase in the frequency, duration and severity of droughts ^6^. These climatic changes are fundamentally reshaping Mediterranean ecosystems distribution.

In the midst of these changes, the dynamics of forest cover are significantly influenced by succession processes triggered by land abandonment, which are currently having a greater impact than climate change ^7^. Recognising this, Spain has prioritised increasing the resilience of forests to drought as a primary objective in the management of protected areas ^8^. Proposed strategies to achieve this include promoting mixed stands and the resilient species to drought, and reducing tree density to decrease competition for water and nutrients during dry periods ^7, 9– 11^

However, a key aspect closely aligned with these proposed strategies is the recognition of the contribution of floristic composition to forest development and conservation.

In this context, the Orchidaceae family stands out as a group of significant biological, ecological and conservation interest. Despite its mainly tropical distribution, approximately 100 species and subspecies of orchids have been documented in the Iberian flora ^12– 17^, with endemic orchids representing 0.7% of the total number of Iberian endemic taxa. This highlights the importance of considering the diversity of the flora present in efforts to increase the resilience of forests and ensure their long-term conservation.

The Orchidaceae family is one of the richest in species (about 20,000), but it is also one of the most threatened in the world by collecting and habitat destruction. So much so that orchids are considered flagship species for plant conservation worldwide ^18^. Many orchids are rare and narrowly distributed in specific habitats ^19– 22^, possibly due to their mycorrhizal specificity, pollinator specialisation and germination limitation ^23, 24^.

Recent research has made significant progress in elucidating the distribution patterns and ecological requirements of endemic orchids in the Iberian Peninsula and the Balearic Islands. Studies by De la Torre ^25^, Pereira *et al*. ^26^ have identified distinct biogeographical regions and hotspots of endemism for orchid taxa, highlighting the importance of localised conservation efforts. Endemic taxa, with their high evolutionary importance and vulnerability to extinction, are priority targets for conservation efforts at different scales ^27^. Previous studies in endemic taxa have provided valuable baseline data such as IUCN categories ^28^, floristic studies ^29^ but there remains a need for more comprehensive biogeographical characterisations and in-depth analyses of the ecological niches of these species.

In this context, detailed biogeographical knowledge of these species and their corresponding cartographic representation is a fundamental tool for their conservation ^30^.

According to Rivas-Martínez *et al*. (2017), Biogeography is the science that studies the distribution of species, communities, habitats, biocoenoses and natural ecosystems on Earth, and the relationships between them. It considers the distribution areas of taxa and syntaxa (chorology), and combines information from other natural sciences (geography, botany, zoology, soil science, bioclimatology, geology, etc.), in order to establish a hierarchical biogeographic classification of the planet territories.

The main typological units in descending rank are kingdom, region, province, sector, district, country, landscape cell and tesela ^31– 35^ ( Table 1). Terrestrial biogeography has been linked to phytogeography due to the value of vascular plant species and communities in defining and delimiting the Earth.

**Table 1.** Typology of biogeographical units in ascending order.

Biogeographical Units	Definition
**Tesela**	Geographical area of greater or lesser extent, ecologically homogeneous, it has only a single type of potential natural vegetation.
**Landscape cell**	Constituted by a mosaic of teselas, it is assembled by networks of geosigmeta based on the geomorphology and the soils of the territory.
**Country**	A large and clearly delimited geographic territory that has a rich set of landscape cells and its own topographical geosigmeta.
**District**	A group of biogeographical countries characterised by a high number of differential species and even endemic taxa, which distinguishes it from neighbouring areas. It also includes units such as associations, series and geoseries that are not present in neighbouring districts.
**Sector**	A group of biogeographical districts forming a largescale geographical unit with its own endemic taxa, associations and vegetation series, as well as original topographic and geoclinosequential (altitudinal zonation) geoseries.
**Province**	It is a large geographical area that contains numerous endemisms and unique species (its own subelement), as well as particular macroseries
**Region**	It comprises a vast area made up by a group of biogeographical provinces, each with its own regional floristic element, including endemic species, genera or even families.
**Kingdom**	It is the supreme unit of biogeography, deals with the origin of the flora and fauna, as well as the origin of the major continents, orogenies and particular macrobioclimates.

The names of the biogeographical units are derived from existing place names, which have been assigned using a standardised methodology for cartography, particularly in Spain. They are considered to be congruent with the area they are intended to represent.

It should be emphasised that the delimitation of biogeographical units can only be accurately determined by their diagnosis and corresponding maps. All areas, with the exception of the teselas, must be contiguous by land, lake or sea routes and include all the small orographic accidents and lithological diversity in the area. Sometimes, neighbouring areas invade biogeographical regions, creating "islands", which often occur in areas with different lithologies or near regional or provincial borders. These islands may have a lower typological independence than the area they intrude, which depends on their originality, floristic richness, phytocoenosis and surface area.

One of the criteria traditionally used for the recognition and delimitation of biogeographical units, as well as for the determination of their entity, is the inclusion of information on the geographical distribution of those taxa (families, genera, species, and subspecies) that are narrowly restricted to a particular area, up to the rank of biogeographical province. In this line, endemisms have been successfully used to define and delimit the chorological or biogeographical units (provinces, sectors and districts), and they form part of the phytogeographical subelement that characterises them.

According to the proposal of Rivas-Martínez *et al*. (2017), the territory of the Iberian Peninsula and the Balearic Islands share two biogeographical regions: the Eurosiberian and the Mediterranean, both included in the Holarctic Kingdom. The numerical synthesis of biogeographical units is displayed in Table 2.

**Table 2.** Number of biogeographical units in the Iberian Peninsula and Balearic Islands.

Biogeographic unit	Eurosiberian Region	Mediterranean Region	Total
**Subregions**	2	1	3
**Provinces**	2	6	8
**Subprovinces**	6	10	16
**Sectors**	12	37	49
**Districts**	64	182	264

Motivated by the need for a more holistic understanding of the distribution, ecology and conservation status of endemic orchids in the region, this study aims to fill existing knowledge gaps and contribute to the conservation and management of these unique taxa within the Iberian Mediterranean forests. By integrating biogeographical mapping, bioclimatic analysis, and phytosociological description, we aim to provide a comprehensive assessment of the distribution patterns, ecological preferences and conservation status of endemic orchid species, including *Epipactis cardina*, *Ophrys bertolonii* subsp. *balearic*a, *Ophrys speculum* subsp. *lusitanica*, *Orchis cazorlensis* and *Serapias perez-chiscanoi*.

Three aspects are present as novelty: 1) the biogeographical characterisation, a set of rules and criteria are proposed that allow us to assign in a simple, objective and universal way the correct biogeographical epithet that corresponds to the taxon according to its distribution area. 2) The current conservation status of the aforementioned species including their phytosociological behaviour. 3) The bioclimatic characterisation, which defines both the qualitative and quantitative requirements for these species, endemic to the Iberian Mediterranean grasslands and forests.

## Methods

### Study area

The Iberian Peninsula (Portugal, Spain and Andorra) including the Balearic Islands (a total of 4992 km ^2^) was studied.

### Data compilation

Five taxa (three species and two subspecies) considered Iberian and/or Balearic endemisms ^11^ were studied ( Table 3).

**Table 3.** Studied taxa.

Genus	Species / Subespecies	Authorship
***Epipactis***	*E. cardina*	Benito & C.E. Hermos. in Estud. Mus. Ci. Nat. Álava 13: 108–109 (1998)
***Ophrys***	*O. bertolonii* subsp. *balearica*	(P. Delforge) L. Sáez & Rosselló in Fl. Montiber. 7: 88 (1997)
***Ophrys***	*O. speculum* subsp. *lusitanica*	O. Danesch & E. Danesch in Orchidee (Hamburg) 20: 21 (1969)
***Orchis***	*O. cazorlensis*	Lacaita in Cavanillesia 3: 35 (1930)
***Serapias***	*S. perez-chiscanoi*	Acedo in Anales Jard. Bot. Madrid 47: 510 (1990)

These five species were specifically chosen because they are Iberian endemics, making them ideal candidates to validate the biogeographical methodology and demonstrate the suitability of the Iberian Peninsula and the Balearic Islands as a representative study area for this type of research. To obtain plant occurrence records for each taxon, a search was conducted using the Global Biodiversity Information Facility ^36– 40^. This database stands as the primary source of Iberian plant distribution data due to the inclusion of all plant records from the Spanish Plant Information System ANTHOS ( http://www.anthos.es). Additionally, we utilised the Flora-On database ( https://flora-on.pt/) to supplement the information.

All presence points were compiled into an Excel file. Records with uncertain origin and undefined locations were removed, following the distribution criteria outlined in *Flora Iberica* ^13^. Subsequently, the Excel data was exported to ArcGIS software for mapping the species' distribution points. The final records underwent manual verification by our team of botanical experts to ensure accuracy.

### Mapping generation

To generate the biogeographical maps for each taxon, we used the shape file from the Biogeographic Map at the District Level of the Iberian Peninsula and Balearic Islands (Annex 1 in extended data) as our basis. his shape file was created through previous research ^41^ conducted with ArcGIS 10.8 software ^42^. The occurrence points of each taxon were exported as a point shape file and overlaid with the polygon shape file of biogeographical districts in ArcGIS 10.8 software.

The detailed biogeographical final map for each taxon was obtained by colouring the districts that match the occurrence points of the taxa, using the geostatistical tool selection by location. This method allows for a quick and easy visualisation of the presented information.

### Proposed criteria and biogeographical characterization

Biogeographical terminology is often used very loosely and therefore with little specificity. Although sometimes the use of a macro-territorial term may be sufficient, when it comes to biogeographical nomenclature applied to an endemism, a more specific term is usually required. Widely used terms such as Eurosiberian, Circummediterranean, Western Iberian, etc. provide rather limited information on the distribution area of endemic taxa such as those we are dealing with here.

For this reason, we propose a set of rules and criteria that will allow us to assign in a simple, objective, and universal way the specific biogeographical epithet that corresponds to the taxon according to its more detailed area of distribution. On the basis of this proposal, the biogeographical characterisation of each taxon is presented.

### Bioclimatic range

For establishing the qualitative and quantitative diagnosis, we have calculated the bioclimatic parameters and indices for each location of each taxon studied, following the Worldwide Bioclimatic Classification System ^43^. Monthly temperature and precipitation values were obtained from the WorldClim database ( www.worldclim.org) from 1970–2019 ^44^. For each taxon, we calculated the maximum, minimum and interquartile range (Q1 and Q3) of each variable examined. To present these results in an easily understandable format, we used box-whisker plots. A box-whisker plot is a visual representation of descriptive statistical data that shows the minimum, first quartile, median, third quartile and maximum values. The top of the box symbolises the third quartile, while a horizontal line near the centre of the rectangle indicates the median. The base of the box represents the first quartile, and in the edge of the vertical lines extending from the top and bottom of the rectangle represent the maximum and values minimum respectively ^45, 46^.

### Phytosociological adscription and conservation status

In order to know the type of habitat in which each taxon occurs, a bibliographic review was carried out to describe their phytosociological behaviour at the level of class, order and alliance when possible ^32– 34, 47^. In addition, the information has been completed with data from the Iberian and Macaronesian Vegetation Information System (SIVIM database, www.sivim.info) ^48, 49^. For taxa for which no phytosociological information was found, an adscription is proposed on the basis of the habitat characteristics reported in the literature ^13, 50– 52^, and data of Interactive Flora of Portugal (FLORA-ON database, https://flora-on.pt/).

Furthermore, the information for each taxon is completed with up to date data on its legal protection status and threat category, if available, (legal provisions and Red Lists and Red Books at regional, national or international levels) obtained from: the PHYTEIA database (Information System on the Protected and Threatened Flora of Spain, module of ANTHOS, http://www.anthos.es); the DRIADA web ( https://www.conservacionvegetal.org/driada/), that includes all the Spanish vascular flora taxa listed in the Spanish Catalogue of Threatened Species, besides the List of Wild Species under special protection regime, and the regional catalogues; and the Red List of the vascular flora of continental Portugal ( https://listavermelha-flora.pt/).

## Results and discussion

**Figure 1.**
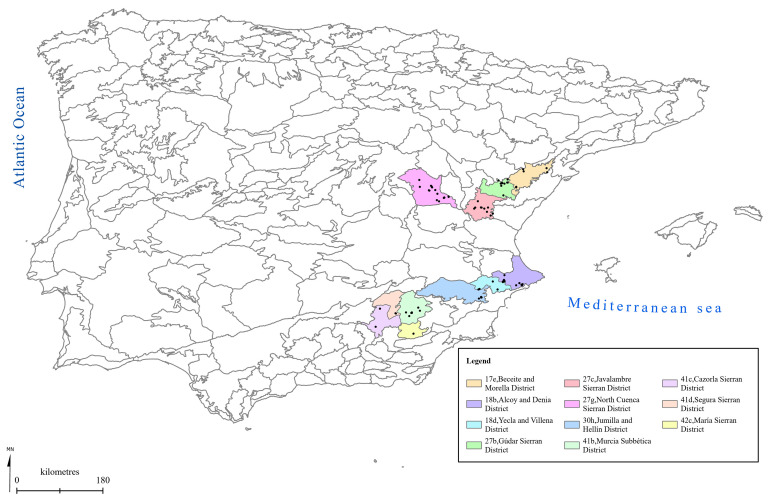
Biogeographical map of *Epipactis cardina*. (●) Occurrence records.

**Figure 2.**
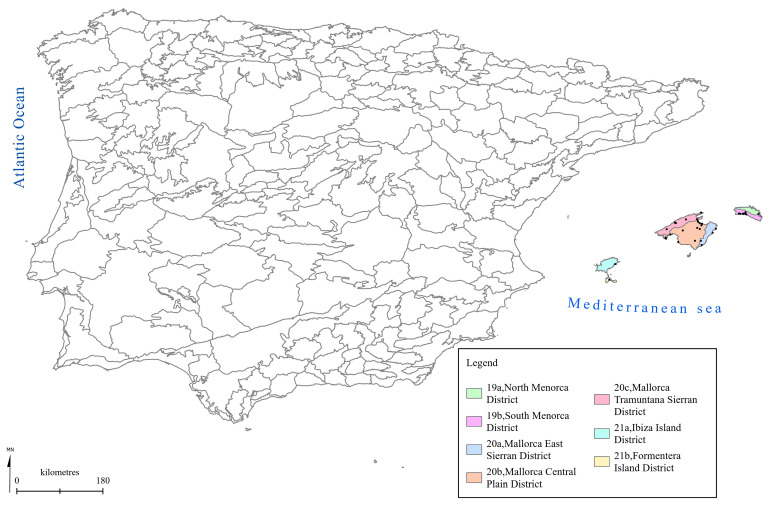
Biogeographical map of *Ophrys bertolonii* subsp. *balearica*. (●) Occurrence records.

**Figure 3.**
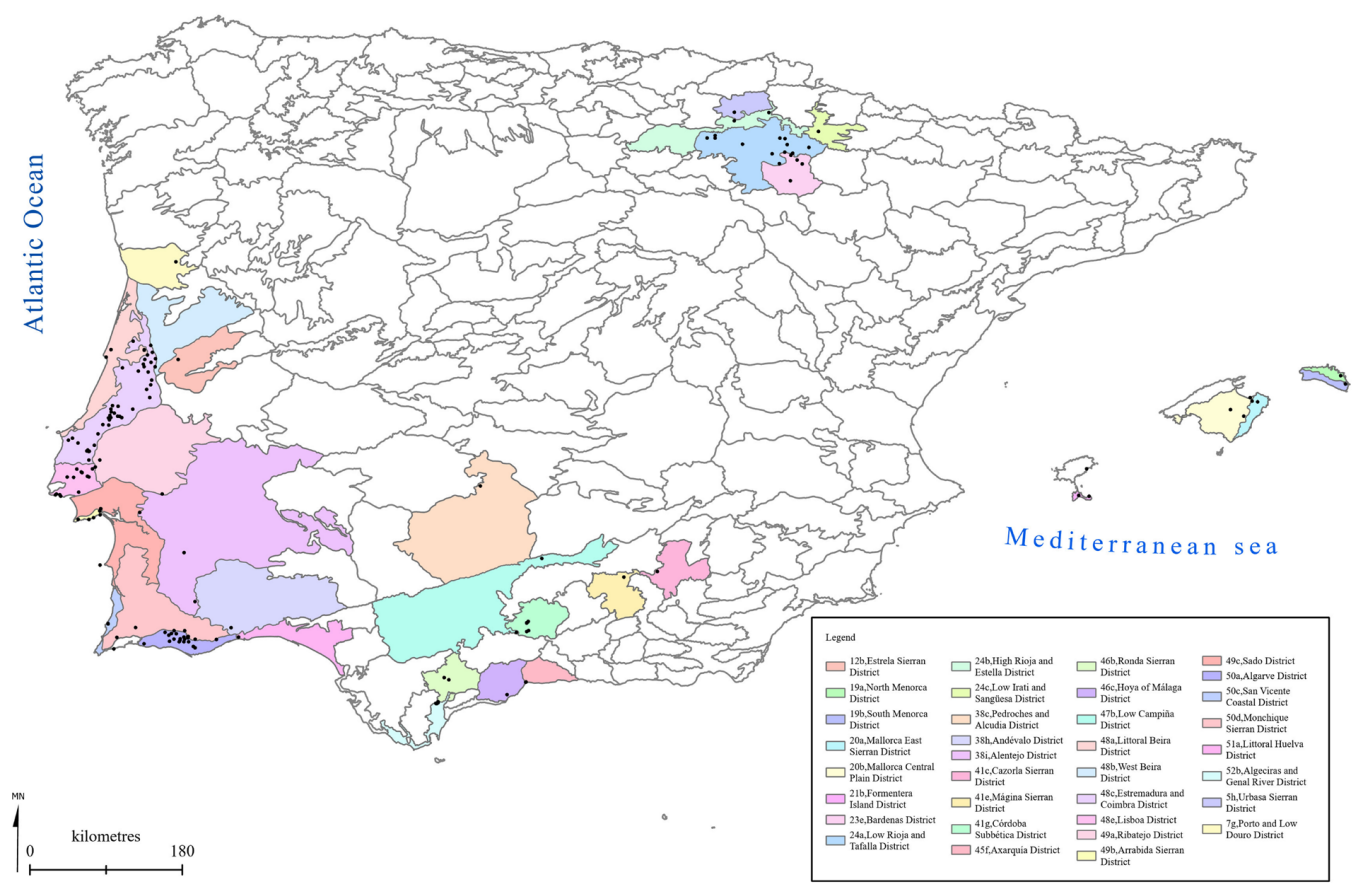
Biogeographical map of *Ophrys speculum* subsp. *lusitanica*. (●) Occurrence records.

**Figure 4.**
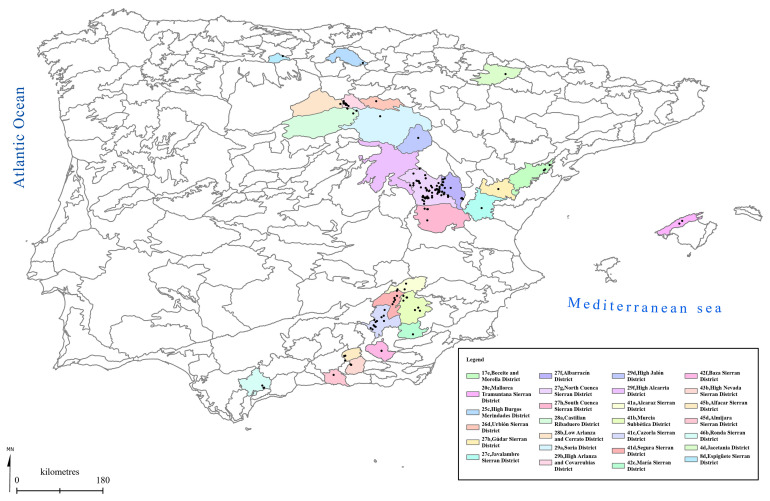
Biogeographical map of *Orchis cazorlensis*. (●) Occurrence records.

**Figure 5.**
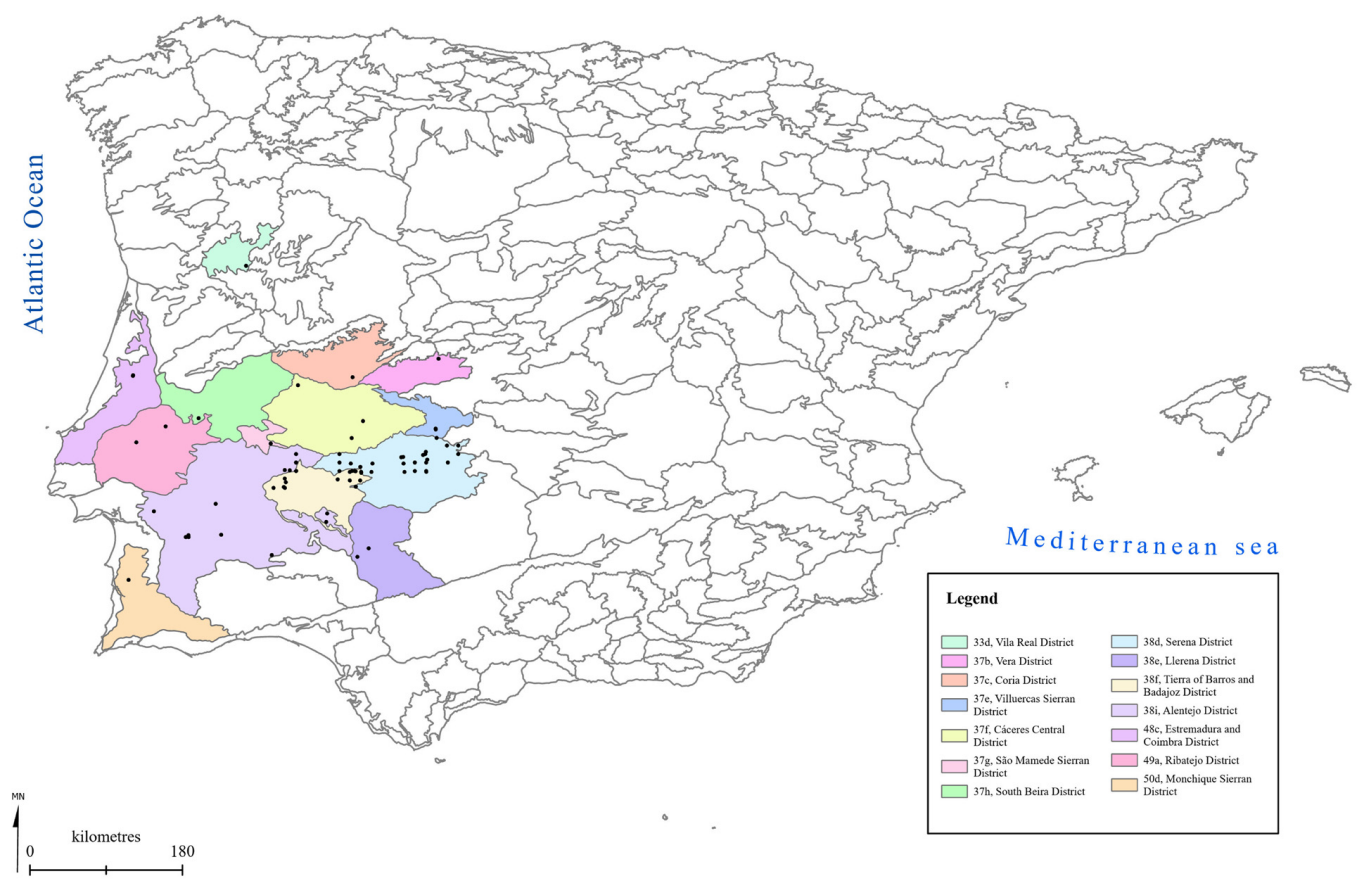
Biogeographical map of *Serapias perez-chiscanoi*. (●) Occurrence records.

Figure 1 to Figure 5 show the biogeographical maps obtained for the taxa studied: *Epipactis cardina* ( Figure 1), *Ophrys bertolonii* subsp. *balearica* ( Figure 2), *O. speculum* subsp. *lusitanica* ( Figure 3), *Orchis cazorlensis* ( Figure 4), and *Serapias perez-chiscanoi* ( Figure 5). The legend shows the names of the biogeographical districts in which the plant occurs. The occurrence records obtained for each plant are also given.

### Proposed criteria and biogeographical characterisation

We propose the following rules and criteria for the biogeographical characterisation of the taxa:

1. The biogeographical characterisation epithet of a taxon shall be formed from the names of the biogeographical units in which that taxon occurs.
2. The epithet shall always be formed from the lowest possible biogeographical unit in which the taxon occurs.
3. If the taxon occurs in 1, 2 or 3 of those lower ranked units, the epithet is formed using the names of those units.
4. If the taxon is present in more than 3 of these lower-level units, the name of the immediately higher-level unit or units is taken.
  a. If the taxon occurs in more than 3 districts, the epithet is formed from the name of the corresponding sector(s).
  b. If the taxon occurs in more than 3 sectors, the epithet is formed from the name of the corresponding subprovince or subprovinces.
  c. If the taxon occurs in more than 4 subprovinces, the epithet is formed from the name of the corresponding province or provinces.
  d. If the taxon occurs in more than 5 provinces, the epithet shall be formed from the name of the corresponding subregion(s).
  e. If the taxon occurs in more than 3 subregions, the epithet shall be formed with the name of the corresponding region or regions.
5. In any case, if the units used to form the epithet are the only ones comprising the immediately superior biogeographical unit, the name of that superior unit shall be taken.
6. In the case of points 4.b and 4.c, if the name of the subprovince or province used to form the epithet is derived from the presence of the taxon in a single district or sector thereof, the name of that district or sector should be given in brackets.
7. In the absence of an auxiliary unit (subprovince or subregion), the biogeographical unit at the immediately higher level should be considered.
8. The names used to form the epithet should be adjectival, separated by commas and arranged in alphabetical order.

This proposal follows the biogeographical units established by Rivas-Martínez *et al.* (2017). which provides not only chorological data but also insights into various environmental characteristics such as lithology, soils, successional dynamics and land use. While this study focuses primarily on chorological aspects, future research could be expanded to include these additional dimensions to enrich the ecological and conservation framework ^53^. It is necessary that the distribution territory of the taxa is thoroughly studied, both floristically and phytosociologically, and that there is a detailed cartography defining the boundaries of each biogeographical unit, which is the case in the Iberian Peninsula.

On the basis of this proposal and according to the biogeographical maps obtained, the biogeographical characterisation of the taxa studied ( Table 4) was carried out.

**Table 4.** Biogeographical characterisation of the studied endemic orchids.

Taxon	Biogeographical characterisation	Criteria applied
***Epipactis cardina***	Betic, Castilian, Oroiberian, Valencian endemism	4b, 6, 7
***Ophrys bertolonii*** **subsp.** ***balearica***	Balearic endemism	4a, 5
***Ophrys speculum*** **subsp.** ***lusitanica***	Betic, coastal Lusitanian-western Andalusian, European Atlantic, western Iberian Mediterranean (Mariánica Range) endemism	4c, 6
***Orchis cazorlensis***	Betic, central Iberian Mediterranean, European Atlantic (Espigüete Sierran), Pyrenean (Jacetania), Valencian-Provençal-Balearic endemism	4c, 6
***Serapias perez-chiscanoi***	Coastal Lusitanian- (Lusitanian Douro) western Andalusian, Lusitanian-Extremaduran endemism	4b, 5

### Bioclimatic characterisation

The box plots obtained for the different parameters and bioclimatic indices analysed for each endemism are presented in this part.

The compensated thermicity index and the positive temperature make it possible to define the thermal component of the bioclimatic zone.

**Figure 6.**
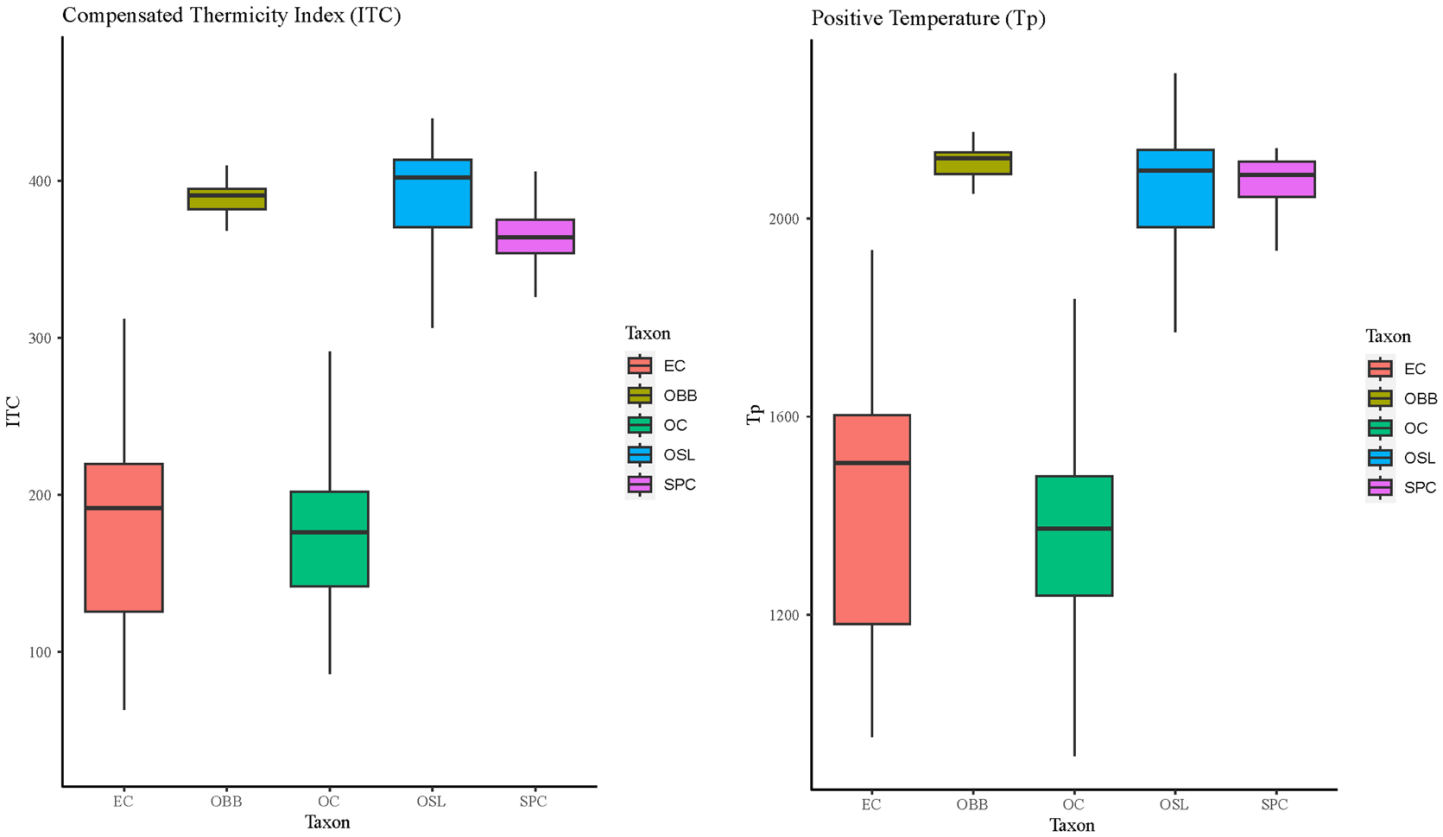
Boxplots for Compensated Thermicity Index (Itc) and Positive Temperature (Tp). Abbreviations: EC: *Epipactis cardina*, OBB: *Ophrys bertolonii* subsp. *balearica*, OC: *Orchis cazorlensis*, OSL: *Ophrys speculum* subsp. *lusitanica*, SPC: *Serapias perez-chiscanoi*.

The thermotype boundary for the taxa studied in this research showed two distinct groups ( Figure 6). Firstly, both *Epipactis cardina* and *Orchis cazorlensis* reached a similar range of thermotype levels: mesomediterranean and supramediterranean, although *O. cazorlensis* is mainly developed at the supramediterranean level. The other three species, *Ophrys bertoloni* subsp. *balearica*, *Ophrys speculum* subsp. *lusitanica* and *Serapias perez-chiscanoi*, showed higher thermotype levels from thermomediterranean to inframediterranean.

**Figure 7.**
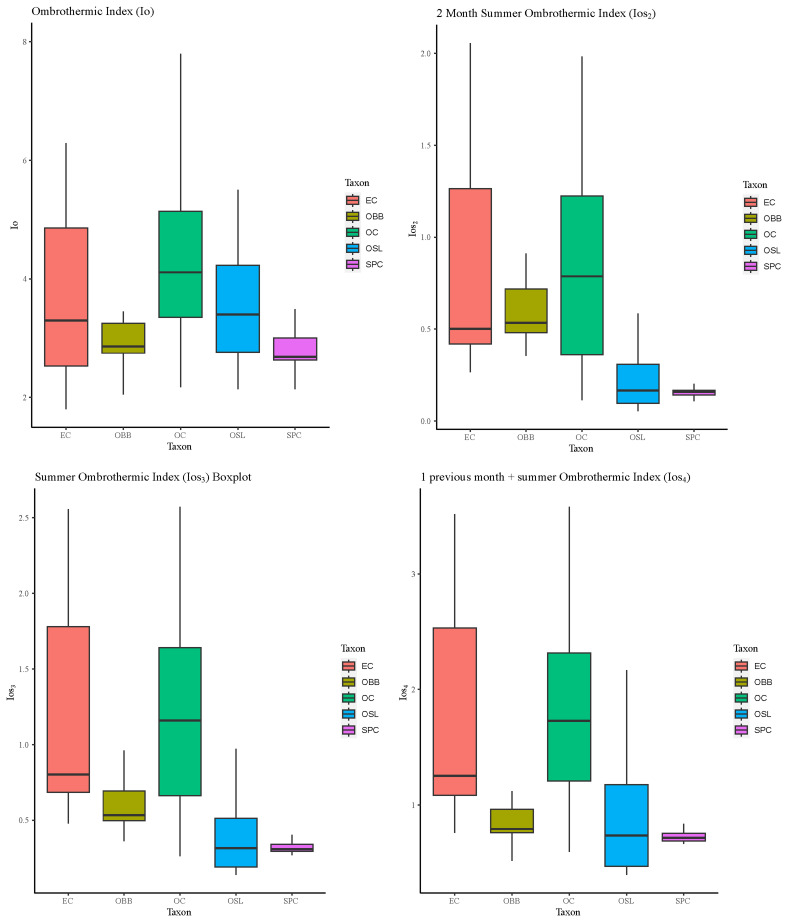
Boxplots of annual ombrothermic index (Io); ombrothermic index of the hottest two months of the summer quarter (Ios2); ombrothermic index of the summer quarter (Ios3) and ombrothermic index of the four-month period (Ios4) for each one the taxon studied. Abbreviations: EC: *Epipactis cardina*, OBB: *Ophrys bertolonii* subsp. *balearica*, OC: *Orchis cazorlensis*, OSL: *Ophrys speculum* subsp. *lusitanica*, SPC: *Serapias perez-chiscanoi*.

If we focus on the rainfall for plants, the annual precipitation rate is more important than the amount. The annual ombrothermic index (Io) represents the ratio of the average rainfall in millimetres to the total rainfall in months where the temperature is above freezing, as the root water absorption processes of plants are hindered below this temperature. In this research, results have shown that the species that exhibited the widest range of ombric tolerance was *Orchis cazorlensis*, spanning from dry to humid environments ( Figure 7). This nuance supports with the information provided in the available *Flora Iberica* data, which indicates locations ranging from 900 to 1850 m in altitude. Additionally, on the website of Flora Protegida ( http://www.floraprotegida.es), it is defined solely as supramediterranean subhumid, in this regard this research gives more detail.

In addition, the summer ombrothermic indices are fundamental as they discriminate between Temperate and Mediterranean bioclimatic areas. The species studied have distinctly Mediterranean macrobioclimate characteristics, except in some places where *E. cardina* develops, which are Temperate macrobioclimate. This is consistent with the habitat description in dry mountain locations but some of the points achieve more precipitation and less temperature as can be seen in the values of Ios _2_^54, 55^.

The species with the most restrictive bioclimatic range is *S. perez-chiscanoi* ( Figure 7), it grows in a strict summer drought, and it does not usually tolerate constant waterlogging for long periods of time. In addition, can only be found in low elevation ^56^. Previous research establishes this species as Upper Mesomediterranean thermotype and Lower subhumid ombrotype ^57^. Our research establishes a more detail bioclimatic belts. Furthermore, if climatic conditions persist in the future as indicated by the IPCC ^58, 59^, this species could see its populations affected by climate change.

**Figure 8.**
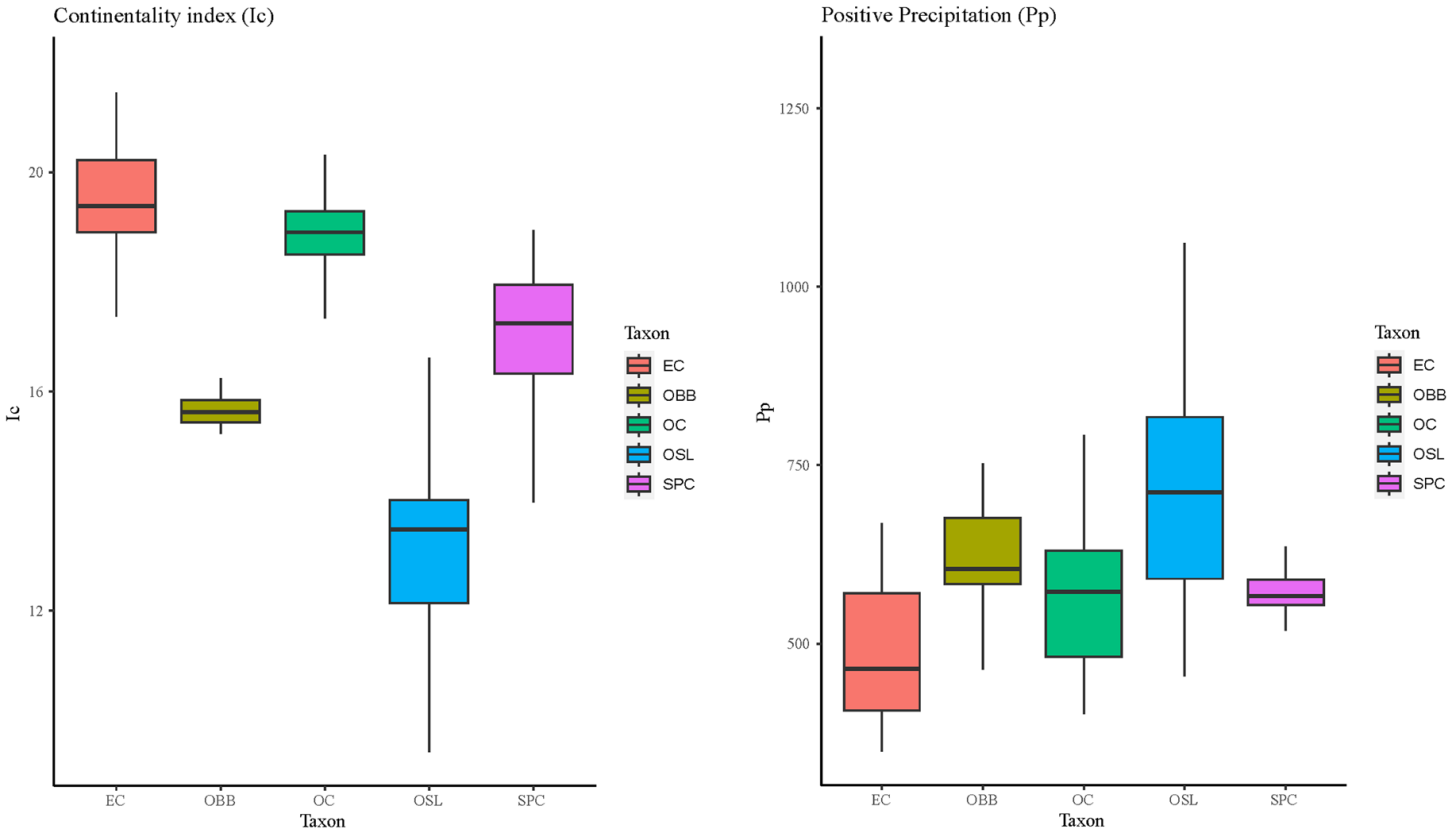
Boxplots of Continentality index (Ic) and Positive precipitation (Pp) for each one of the taxon studied. Abbreviations: EC: *Epipactis cardina*, OBB: *Ophrys bertolonii* subsp. *balearica*, OC: *Orchis cazorlensis*, OSL: *Ophrys speculum* subsp. *lusitanica*, SPC: *Serapias perez-chiscanoi*.

According to Continentality index, that reflects the amplitude of the annual oscillation of temperature ^43^, results show ( Figure 8) that the more continental species is *E. cardina* and the more oceanic one is *Ophrys speculum* subsp. *lusitanica*.

Finally, the qualitative bioclimatic characterisation was determined according to the bioclimatic classification outlined by Rivas-Martínez *et al*. (2011). As indicated in previous research ^60^, we have specified the most common isobioclimate in which a taxon develops. Brackets are used to denote situations where a taxon might occur in another isobioclimate on an ad hoc basis. Quantitative diagnoses include the lowest value, the interquartile range (in bold and brackets) and the highest value for each measured variable.

***Epipactis cardina***

Mediterranean pluviseasonal oceanic and Temperate oceanic submediterranean semicontinental supramediterranean and supratemperate lower dry to upper subhumid

(Temperate oceanic semicontinental lower supratemperate from upper subhumid to lower humid)

T _avg_: 7.8 **(9.8-13.3)** 16.1; T _max_: 5.90 **(16.9-20.3)** 23.0; T _min_: -5.3 **(2.7-6.4)** 9.3; Tp: 953.1 **(1181.0-1603.7)** 1936.0; Pp: 349.1 **(405.9-571.6)** 668.9; Itc: 63.4 **(123.8-219.7)** 312.3; Ic: 17.4 **(18.9-20.2)** 21.5; Io: 1.8 **(2.5-4.9)** 6.3; Ios _2_: 0.3 **(0.5-1.3)** 2.1; Ios _3_: 0.5 **(0.7-1.8)** 2.6; Ios _4_: 0.8 **(1.1-2.5)**3.5.

***Ophrys speculum subsp. lusitanica***

Mediterranean pluviseasonal oceanic subhyperoceanic to semicontinental lower thermomediterranean to upper mesomediterranean lower dry to upper humid

T _avg_: 14.8 **(16.5-17.8)** 19.1; T _max_: 19.5 **(21.3-23.2)** 26.0; T _min_: 7.9 **(11.2-12.7)** 13.8; Tp: 884.0 **(1046.0-1386.0)** 1977.0; Pp: 454.0 **(590.8-822.5)** 1303.0; Itc: 261.0 **(370.5-413.5)** 440.0; Ic: 9.4 **(12.1-14.0)** 20.4; Io: 2.1 **(2.8-4.3)** 7.0; Ios _2_: 0.1 **(0.1-0.3)** 0.9; Ios _3_: 0.1 **(0.2-0.5)** 1.3; Ios _4_: 0.4 **(0.5-1.2)** 2.2.

***Ophrys bertolonii subsp. balearica***

Mediterranean pluviseasonal oceanic and Mediterranean xeric oceanic weak euoceanic lower thermomediterranean to lower mesomediterranean upper semiarid to lower subhumid

T _avg_: 14.9 **(17.4-17.8)** 18.9; T _max_: 20.1 **(21.6-23.0)** 24.4; T _min_: 10.5 **(12.4-13.6)** 14.3; Tp: 1785.0 **(2086.9-2135.7)** 2266.6; Pp: 412.3 **(577.3-679.5)** 752.8; Itc: 303.6 **(380.9-395.4)** 417.8; Ic: 15.2 **(15.4-15.9)** 16.8; Io: 1.8 **(2.7-3.2)** 4.2; Ios _2_: 0.4 **(0.5-0.7)** 0.9; Ios _3_: 0.4 **(0.5-0.7)** 1.0; Ios _4_: 0.5 **(0.8-1.0)** 1.4.

***Orchis cazorlensis***

Mediterranean pluviseasonal oceanic and Mediterranean pluviseasonal continental and Temperate oceanic submediterranean weak euoceanic to weak subcontinental mesomediterranean to supramediterranean and upper supratemperate lower dry to upper humid

T _avg_: 7.4 **(10.3-12.4)** 15.4; T _max_: 13.6 **(16.8-18.6)** 22.3; T _min_: 0.2 **(3.5-5.9)** 10.2; Tp: 915.3 **(1238.5-1485.8)** 1845.0; Pp: 401.3 **(480.4-626.8)** 966.0; Itc: 35.8 **(141.4-202.1)** 310.6; Ic: 15.2 **(18.5-19.3)** 21.8; Io: 2.1 **(3.3-5.2)** 8.0; Ios _2_: 0.1 **(0.4-1.2)** 2.0; Ios _3_: 0.3 **(0.7-1.6)** 2.6; Ios _4_: 0.6 **(1.2-2.3)** 3.6.

***Serapias perez-chiscanoi***

Mediterranean pluviseasonal oceanic weak semihyperoceanic weak semicontinental and upper inframediterranean to upper mesomediterranean lower dry to lower subhumid.

T _avg_: 13.0 **(17.0-17.62)** 17.8; T _max_: 17.5 **(23.0-24.2)** 24.6; T _min_: 2.8 **(4.3-5.1)** 11.4; Tp: 1558.0 **(2043.1-2114.6)** 2141.5; Pp: 413.7 **(554.0-589.6)** 1191.0; Itc: 242.3 **(354.2-375.4)** 470.5; Ic: 11.5 **(16.4-18.0)** 18.9; Io: 2.1 **(2.6-3.0)**7.64; Ios _2_: 0.1 **(0.1-0.2)** 0.8; Ios _3_: 0.3 **(0.3-0.4)** 1.45; Ios _4_: 0.7 **(0.7-0.8)** 2.43.

### Phytosociological adscription and conservation status

The phytosociological description of the taxa studied is given in Table 5. Information of legal protection status and threat category is shown in Table 6.

**Table 5.** Phytosociological adscription of the studied endemic orchids.

Taxon	Phytosociological adscription	Ecology
***Epipactis cardina***	*Quercetea ilicis* Br.-Bl. ex A. & O. Bolòs 1950 *Rosmarinetea officinalis* Rivas-Martínez, T.E. Díaz, F. Prieto, Loidi & Penas 2002	Pine forests and sclerophyllous scrubland, on dry and preferably basic soils.
***Ophrys bertolonii*** **subsp.** ***balearica***	*Querco rotundifoliae-Oleion sylvestris* Barbero, Quézel & Rivas- Martínez in Rivas-Martínez, Costa & Izco 1986 ( *Quercetea ilicis* Br.-Bl. ex A. & O. Bolòs 1950)	Mediterranean Scrubland and forest glades
***Ophrys speculum*** **subsp.** ***lusitanica***	*Rosmarinetea officinalis* Rivas-Martínez, T.E. Díaz, F. Prieto, Loidi & Penas 2002	Scrub and grassland on stony soils
***Orchis cazorlensis***	*Quercetea ilicis* Br.-Bl. ex A. & O. Bolòs 1950 *Rosmarinetea officinalis* Rivas-Martínez, T.E. Díaz, F. Prieto, Loidi & Penas 2002	Mediterranean Scrubland and forest glades
***Serapias perez-*** ***chiscanoi***	*Holoschoenetalia vulgaris* Br.-Bl. ex Tchou 1948 ( *Molinio caeruleae-* *Arrhenatheretea elatioris* Tüxen 1937) *Agrostion castellanae* Rivas Goday ex Rivas-Martínez, Costa, Castroviejo & E. Valdés 1980 ( *Stipo giganteae-Agrostietea castellanae* Rivas-Martínez, Fernández González & Loidi 1999)	Meadows and pastures with certain soil moisture

**Table 6.** Conservation status and threat category of the studied endemic orchids.

Taxon	Legal protection status	IUCN category
***Epipactis *** ***cardina***	Protected at regional level by Murcia legislation with the category “Of Special Interest” ^62^	Included in the regional Red Lists of Murcia ^63^ and Catalonia as Data Deficient (DD) ^64^
***Ophrys *** ***bertolonii*** **subsp.** ***balearica***	Not legally protected	Included in the regional Red List of the Balearic Islands as Least Concern (LC) ^65^
***Ophrys *** ***speculum*** **subsp.** ***lusitanica***	Protected at regional level by Andalusian legislation as “Vulnerable” ^66^	Included in the regional Red List of Andalusia as Data Deficient (DD) ^67^
***Orchis *** ***cazorlensis***	Protected at regional level by the legislation of the Balearic Islands ("In danger of extinction" (In danger of extinction) ^68, 69^, Catalonia ("In danger of extinction") ^70^, Castilla y León ("Of preferential attention") ^71^, Murcia ("Vulnerable") ^62^ and the Basque Country ("In danger of extinction") ^72^	Included in the regional Red Lists of Baleares as Vulnerable (VU) ^73^, Catalonia as Critically Endangered (CR) ^73^, Murcia as Vulnerable (VU) ^74^ and Basque Country as Critically Endangered (CR) ^75^
***Serapias perez*** ***-chiscanoi***	Protected at regional level by Extremadura legislation with the category of “In danger of extinction” ^76, 77^	Included in the Red List of Spanish Flora as Near Threatened (NT) ^78^, and in the Red List of the vascular flora of continental Portugal as Endangered (EN) ^79^

These orchids live mainly in Mediterranean forests and scrub clearings of the classes *Quercetea ilicis* and *Rosmarinetea officinalis*, and only *Serapias perez-chiscanoi* has higher soil moisture requirements and is found in meadows and pastures of *Molinio-Arrhenatheretea* and *Stipo-Agrostietea*.

These plant communities are included in Annex I of Directive 92/43/EEC ^61^, which obliges Member States to establish a network of special areas that guarantees their conservation or restoration to a favourable state ^61^, with the following codes of Habitats of Community Interest (HICs): 9340- *Quercus ilex* and *Q. rotundifolia* forests, 5330-Mediterranean scrub and thymes, 6420-Mediterranean tall humid grasslands of the *Molinio-Holoschoenion*, and 6220*-Mediterranean xerophytic perennial and annual grasslands. The last one has the category of Priority Natural Habitat. *Molinio-Holoschoenion*, and 6220*-Mediterranean xerophytic perennial and annual grasslands. The last one has the category of Priority Natural Habitat.

All taxa are legally protected at regional level apart from *Ophrys bertolonii* subsp. *balearica*. The orchids *Orchis cazorlensis* and *Serapias perez-chiscanoi* are listed as endangered (category of "In danger of extinction") in some Spanish territories such as the Balearic Islands, Catalonia, the Basque Country, or Extremadura. None of them are legally protected at national or European level.

Furthermore, all of them are included in a regional or national Red List. However, two of them ( *Epipactis cardina* and *O. speculum* subsp. *lusitanica*) are listed in the DD category, which implies that more knowledge is needed to assign them (or not) a threat category. Others are in non-threatened IUCN categories such as NT ( *S. perez-chiscanoi*) or LC ( *O. bertolonii* subsp. *balearica*) and others are in IUCN threatened categories (VU, EN and CR). Of particular concern is the case of *Orchis cazorlensis* in the Basque Country and Catalonia where it is classified as CR. Only *S. perez-chiscanoi* appears on the national Red Lists of Spain and Portugal, in the latter case with the category EN.

## Conclusions

Detailed mapping and biogeographical characterisation identified specific regions of endemism for each species, providing valuable insights into their distribution patterns. The proposed rules for biogeographical characterisation provided a systematic approach that increased the precision of the definition of their endemic areas. Bioclimatic analyses revealed different climatic preferences, shedding light on their ecological niches and phytosociological patterns. These results lead to the following conclusions:

- The orchids *Epipactis cardina*, *Ophrys bertolonii* subsp. *balearica*, *Ophrys speculum* subsp. *lusitanica*, *Orchis cazorlensis* and *Serapias perez-chiscanoi*, exemplify unique and diverse taxa within the Iberian Peninsula and the Balearic Islands.

- Biogeographical characterisations reveal the complex relationships between these taxa and their respective regions, indicating their adaptability to local environmental conditions. In addition, the biogeographical maps illustrate the specific distribution patterns of these orchids, providing insights into their geographical ranges.

- The proposed criteria for the biogeographical characterisation of taxa are objective, useful and relevant both for endemics from very localised and restricted areas, such as *Ophrys bertolonii* subsp. *balearica*, and for those with a wider distribution, as is the case of *Orchis cazorlensis* and *Ophrys speculum* subsp. *lusitanica.* Understanding their biogeographical distribution and ecological requirements is crucial for their conservation in Mediterranean forests.

- In terms of precipitation, annual ombrothermic index (Io) results highlight the diverse ombric tolerances of these species, with *Orchis cazorlensis* showing the widest range, thriving from dry to humid environments.

- *Serapias perez-chiscanoi* emerges as the most sensitive species, restricted to severe summer drought conditions and lower elevations, making it vulnerable to future climate change impacts as shown by the IPCC scenarios. This sensitivity to climatic factors, including drought, underlines the vulnerability of these orchids, with narrower bioclimatic ranges, to changing climate patterns.

- The habitat of these endemic orchids is mainly linked to Mediterranean scrubs and forests, and to a lesser extent to grasslands and pastures (classes *Quercetea ilicis, Rosmarinetea officinalis*, *Molinio-Arrhenatheretea* and *Stipo-Agrostietea*).

- All the orchids studied are part of the Habitats of Community Interest (HICs) and, in the case of *Serapias perez-chiscanoi*, it is also a Priority Natural Habitat. This reinforces the need to conserve these species and plant communities in which they develop.

- In the case of *Orchis cazorlensis*, despite regional legal protection, its Critically Endangered (CR) status on the Red Lists of certain regions raises alarm about the immediate threats it faces. It is also important to note the case of *Serapias perez-chiscanoi*, listed as Endangered (EN) in the Portuguese Red List. Urgent conservation efforts are therefore essential. In addition, *Epipactis cardina* and *Ophrys speculum* subsp. *lusitanica* are listed as Data Deficient (DD) on the regional Red Lists, requiring immediate comprehensive surveys to determine its population size, habitat health and potential threats.

This research will allow greater prospection to obtain a more reliable knowledge of the species studied in the Mediterranean forests, scrubs and grasslands of the Iberian Peninsula and the Balearic Islands. It is worth noting the support that this type of study can bring together to improve the data available to environmental management professionals. In this regard, the comprehensive biogeographical, phytosociological, and bioclimatic characterisation of these taxa will provide the basis for appropriate decisions on the management measures necessary for their protection and for the possible updating of their conservation status, especially those focused on the conservation of endemic taxa.

## Data Availability

Zenodo: Data for the research: Detailed biogeographical mapping as a useful novel tool for the conservation of endemic taxa: a case of study for Iberian orchids. https://doi.org/10.5281/zenodo.13324273 ^75^, The project contains the following underlying data. File name: RData File name: - Rhistory- File name: DBioclimendem.xlsx File name: - Endem.xlsx File name: Endemismos nuevo boxplot.R File name: Endemismos21.R Data are available under Creative Commons Attribution 4.0 International Zenodo: Data for the research: Detailed biogeographical mapping as a useful novel tool for the conservation of endemic taxa: a case of study for Iberian orchids. https://doi.org/10.5281/zenodo.13324272 ^75^ The project contains the following extended data. File name- Annex1ProyectFloraEndemicDistrict.pdf Data are available under Creative Commons Attribution 4.0 International
