# Supplementary material for: Detailed biogeographical mapping as a useful novel tool for the conservation of endemic taxa: a case of study for Iberian orchids
Source: Open Res Eur. 2025 Jan 30;4:210. Originally published 2024 Oct 2. [Version 2] doi: 10.12688/openreseurope.17737.2 (PMC11809187; doi:10.12688/openreseurope.17737.2)
Supplement: Peer review history

# Reviewer report: Reviewer response for version 2

**DOI:** 10.21956/openreseurope.21041.r50502

**Published:** 2025-02-05

**Authors:** Pablo J. Hidalgo, Andrés V. Pérez-Latorre

**Affiliations:** 1 University of Huelva, Huelva, Spain; 2 Área de Botánica. Dpto. Botánica y Fisiología Vegetal., University of Malaga (Ringgold ID: 16752), Málaga, Andalusia, Spain

Dear Editor, 

 All the amendments and recommendations given to the authors have been properly addressed. The readability of the article has improved and the document is ready for indexing in its present form.

Is the study design appropriate and does the work have academic merit?

Yes

Is the work clearly and accurately presented and does it cite the current literature?

Yes

If applicable, is the statistical analysis and its interpretation appropriate?

Yes

Are all the source data underlying the results available to ensure full reproducibility?

Yes

Are the conclusions drawn adequately supported by the results?

Yes

Are sufficient details of methods and analysis provided to allow replication by others?

Yes

Reviewer Expertise:

Botany, plant sciences, vegetation, biogeography.

We confirm that we have read this submission and believe that we have an appropriate level of expertise to confirm that it is of an acceptable scientific standard.

# Reviewer report: Reviewer response for version 1

**DOI:** 10.21956/openreseurope.19174.r46629

**Published:** 2024-12-17

**Authors:** Pablo J. Hidalgo, Andrés V. Pérez-Latorre

**Affiliations:** 1 University of Huelva, Huelva, Spain; 2 Área de Botánica. Dpto. Botánica y Fisiología Vegetal., University of Malaga (Ringgold ID: 16752), Málaga, Andalusia, Spain

This study, focused on wild orchids, presents a new and interesting tool for the biogeographical characterization of endangered species. This methodology jointly identifies the biogeographical distribution, bioclimatology, and phytosociological classification as well as Habitats 92/43 for species of interest, specifically tested here with five endemic orchids from the Iberian Peninsula and Balearic Islands.

 The concept is innovative, and the study demonstrates its potential as an additional system for species conservation. In addition, detailed bioclimatological and phytosociological data have led to a more robust classification. The proposed criteria for biogeographical classification are well-designed and follow coherent hierarchical steps. This methodology can be easily reproduced for other endangered plant species and even for Habitats of Community Interest, protected by the European Directive. In this context, the 11 biogeographical regions defined by the European Commission could be enhanced with a more detailed classification based on local or regional data, as demonstrated for orchids in the Iberian Peninsula and Balearic Islands in the present study. In summary, data on biogeography, bioclimatology, and phytosociology-habitats are crucial for developing any recovery/conservation plan and/or to improve red lists or books of threatened species. The combination of these data along with a proposed nomenclature for distribution allows for a more accurate understanding of the plant species of interest.

 Regarding the data presented, the structure and format are appropriate; the document is easy to follow, and the conclusions are clear and concise. The structure of the article is correct and readable. Overall, all sections are accurate, and the holistic approach to studying plant distribution is very positive.

 Specific comments:

- Abstract, Background:

I believe that "Many Iberian orchids occur…" is preferable to "Iberian orchids occur…" as not all of them are found in Habitats of Community Interest (HCI). Unfortunately, many species exist outside of HCI and even Natura 2000 areas.

 Habitat 92/43 that generally contains the orchids is explicitly not a type of forest but rather grasslands (it is also a priority habitat): “6210 Semi-natural dry grasslands and scrubland facies on calcareous substrates ( *Festuco-Brometalia*) (* sites with notable presence of orchids).” Throughout the article, it appears that the forest ecosystem in general is considered the most important for this type of plants in Iberia.

- Plain Language Summary:

Caution: The Balearic Islands are part of Spain.

- Introduction:

End of the first paragraph: It would be better to say "…reshaping Mediterranean ecosystem distribution" instead of "…reshaping Mediterranean ecosystem."

 This phrase may lead to an environmental conflict because introduction is usually applied to alien species that are foreign to the natural composition of forests (“…introducing resilient species”): “Proposed strategies to achieve this include promoting mixed stands and the resilience of species, and reducing tree density 7,9.”

 This recent reference does not appear in the work and is important because it partially aligns with its biogeographical objective and is globally relevant: “Yajun Wang et al. 2024. Progress in systematics and biogeography of *Orchidaceae*. Plant Diversity 46 (2024) 425-434.”

 Regarding the use of biogeographic methodology (Rivas Martínez et al., 2007), it should be emphasized as significant in its corresponding section that the biogeographic identification of a species does not only produce chorological data but also assigns properties related to the physical environment, some of which were not studied in this work, such as lithology, soils, succession dynamics, and land use. [Rivas-Martínez, S. et al. (2007) Map of series, geoseries, and geopermaseries of vegetation in Spain. Report on the Potential Vegetation Map of Spain. Part I. Itinera Geobotanica. 17, 5-436.]

 Regarding the phrase: “The names of the biogeographical units are given on the basis of known geographical, orographic or historical terms”. I believe it would be more accurate to state that these are existing place names that have been declined. Place names are assigned through standardized methodology for cartography, at least in Spain.

 Why were these five species chosen? It seems they were selected due to being Iberian endemics; however, this is not clearly stated in the text. Please specify that testing endemics better validates the methodology for using the peninsula as a study area.

 When citing threat status, include abbreviations in the text; e.g., “In danger of extinction” should be noted as (EN).

 The first conclusion may not be necessary since it is implied that endemic orchids represent unique and diverse taxa within the Iberian Peninsula and Balearic Islands: “represent unique and diverse taxa within the Iberian Peninsula and Balearic Islands.”

 Regarding *Serapias*, it seems more appropriate to discuss conditionally that it could be affected by climate change according to IPCC scenarios. However, this effect appears more likely in those orchids with narrower bioclimatic ranges across all studied parameters.

- Methods, Data compilation.

Please could you change "Excel data-base" by "Excel file"? It refers to a spreadsheet program rather than a database management system. In the same paragraph, could you also provide the proper reference for *Flora Iberica*? It is listed as number 11 in the bibliography.

 Regarding the WorldClim database, it is well-known and widely used by the scientific community. However, the authors should include the following reference alongside the link, as proposed in the web site: Fick, S.E. and R.J. Hijmans, 2017. WorldClim 2: new 1 km spatial resolution climate surfaces for global land areas. International Journal of Climatology 37 (12): 4302-4315.

- Phytosociological adscription and conservation status:

In the second paragraph: ‘…regional, national or international levels’ instead ‘…regional, national or international level’.

- Results and discussion:

Please, review some ‘subsp.’ written in italics in the caption of the Results and discussion section.

- Phytosociological adscription and conservation status:

In the third paragraph, please review the last sentence; it seems that something is repeated.

Is the study design appropriate and does the work have academic merit?

Yes

Is the work clearly and accurately presented and does it cite the current literature?

Yes

If applicable, is the statistical analysis and its interpretation appropriate?

Yes

Are all the source data underlying the results available to ensure full reproducibility?

Yes

Are the conclusions drawn adequately supported by the results?

Yes

Are sufficient details of methods and analysis provided to allow replication by others?

Yes

Reviewer Expertise:

Botany, plant sciences, vegetation, biogeography.

We confirm that we have read this submission and believe that we have an appropriate level of expertise to confirm that it is of an acceptable scientific standard, however we have significant reservations, as outlined above.

# Response

**Published:** 2025-01-17

**Authors:** Alejandro González Pérez

**Affiliations:** Biodiversity and Environmental Management (Botany), Universidad de Leon, León, Castile and León, Spain

**Reviewer Comments:**

 This study, focused on wild orchids, presents a new and interesting tool for the biogeographical characterization of endangered species. This methodology jointly identifies the biogeographical distribution, bioclimatology, and phytosociological classification as well as Habitats 92/43 for species of interest, specifically tested here with five endemic orchids from the Iberian Peninsula and Balearic Islands. The concept is innovative, and the study demonstrates its potential as an additional system for species conservation. In addition, detailed bioclimatological and phytosociological data have led to a more robust classification. The proposed criteria for biogeographical classification are well-designed and follow coherent hierarchical steps. This methodology can be easily reproduced for other endangered plant species and even for Habitats of Community Interest, protected by the European Directive. In this context, the 11 biogeographical regions defined by the European Commission could be enhanced with a more detailed classification based on local or regional data, as demonstrated for orchids in the Iberian Peninsula and Balearic Islands in the present study. In summary, data on biogeography, bioclimatology, and phytosociology-habitats are crucial for developing any recovery/conservation plan and/or to improve red lists or books of threatened species. The combination of these data along with a proposed nomenclature for distribution allows for a more accurate understanding of the plant species of interest. Regarding the data presented, the structure and format are appropriate; the document is easy to follow, and the conclusions are clear and concise. The structure of the article is correct and readable. Overall, all sections are accurate, and the holistic approach to studying plant distribution is very positive.

**Author Response:**

 Dear Reviewers, we are very grateful for your thorough evaluation of this paper. Your constructive feedback and suggestions have provided valuable guidance that has significantly improved both the clarity and quality of our work. We have carefully considered each of your comments, which are addressed individually in the blue text below.

**Reviewer Comments:**

 Specific comments:

- Abstract, Background:

I believe that "Many Iberian orchids occur…" is preferable to "Iberian orchids occur…" as not all of them are found in Habitats of Community Interest (HCI). Unfortunately, many species exist outside of HCI and even Natura 2000 areas.

 Habitat 92/43 that generally contains the orchids is explicitly not a type of forest but rather grasslands (it is also a priority habitat): “6210 Semi-natural dry grasslands and scrubland facies on calcareous substrates ( *Festuco-Brometalia*) (* sites with notable presence of orchids).” Throughout the article, it appears that the forest ecosystem in general is considered the most important for this type of plants in Iberia. Thank you, reviewer, we have modified the wording in the text to give importance to grasslands as well.

- Plain Language Summary:

Caution: The Balearic Islands are part of Spain.

**Author Response:**

 Thank you, reviewer, we have changed the text as “grow in certain parts of Spanish Peninsula and the Balearic Islands”

**Reviewer Comments:**

- Introduction:

End of the first paragraph: It would be better to say "…reshaping Mediterranean ecosystem distribution" instead of "…reshaping Mediterranean ecosystem."

 This phrase may lead to an environmental conflict because introduction is usually applied to alien species that are foreign to the natural composition of forests (“…introducing resilient species”): “Proposed strategies to achieve this include promoting mixed stands and the resilience of species, and reducing tree density 7,9.” Authors agreed and we have modified the first two paragraph to address your comments, please see in the final version of the manuscript.

 This recent reference does not appear in the work and is important because it partially aligns with its biogeographical objective and is globally relevant: “Yajun Wang et al. 2024. Progress in systematics and biogeography of *Orchidaceae*. Plant Diversity 46 (2024) 425-434.”

**Author Response:**

 Thank you, reviewer, we have added that new research [30] that was published after we had finished this research.

**Reviewer Comments:**

 Regarding the use of biogeographic methodology (Rivas Martínez et al., 2007), it should be emphasized as significant in its corresponding section that the biogeographic identification of a species does not only produce chorological data but also assigns properties related to the physical environment, some of which were not studied in this work, such as lithology, soils, succession dynamics, and land use. [Rivas-Martínez, S. et al. (2007) Map of series, geoseries, and geopermaseries of vegetation in Spain. Report on the Potential Vegetation Map of Spain. Part I. Itinera Geobotanica. 17, 5-436.]

**Author Response:**

 We appreciate the reviewer's valuable point about the wider implications of biogeographic methodology. In response, we have included a clarification in the relevant section to emphasise that the biogeographic identification of species provides not only chorological data, but also valuable insights into properties of the physical environment, such as lithology, soils, successional dynamics and land use. Although these aspects were beyond the scope of this study, we recognise their importance and potential for future research. A reference to Rivas-Martínez et al. (2007) has been retained to highlight these additional dimensions and their importance.

**Reviewer Comments:**

 Regarding the phrase: “The names of the biogeographical units are given on the basis of known geographical, orographic or historical terms”. I believe it would be more accurate to state that these are existing place names that have been declined. Place names are assigned through standardized methodology for cartography, at least in Spain.

**Author Response:**

 We appreciate the reviewer's comment on the wording. To ensure accuracy and consistency with cartographic standards, we have revised the text to clarify that the names of the biogeographical units are based on existing place names that have been assigned using a standardised methodology for cartography in Spain. The updated wording reflects this point and ensures consistency with established practice.  

**Reviewer Comments:**

 Why were these five species chosen? It seems they were selected due to being Iberian endemics; however, this is not clearly stated in the text. Please specify that testing endemics better validates the methodology for using the peninsula as a study area.

**Author Response:**

 Thank you, we write a sentence in section 2 Methods, to make it clearer as follows: “ *These five species were specifically chosen because they are Iberian endemics, making them ideal candidates to validate the biogeographical methodology and demonstrate the suitability of the Iberian Peninsula and the Balearic Islands as a representative study area for this type of research".*

**Reviewer Comments:**

 When citing threat status, include abbreviations in the text, e.g., “In danger of extinction” should be noted as (EN).

**Author Response:**

 Thank you, authors state not right to abbreviate that term, because that abbreviation is used only when we are talking about IUCN categories (Red Lists/Red Books). EN would be the abbreviation of the IUCN category ‘Endangered’ (it appears for example in the IUCN category column of that table), but it does not correspond to the categories of the legislations, where the full term must be kept (as the meaning is not the same in both cases, ‘Endangered’ of the IUCN (which is abbreviated as EN) is not the same as ‘In danger of extinction’ of the corresponding autonomous legislation). Therefore, they shouldn´t be abbreviated either in the table or in the text. When they can be abbreviated, they already are (column IUCN category of the same table 6 and second paragraph after the table).

**Reviewer Comments:**

 The first conclusion may not be necessary since it is implied that endemic orchids represent unique and diverse taxa within the Iberian Peninsula and Balearic Islands: “represent unique and diverse taxa within the Iberian Peninsula and Balearic Islands.”

 Regarding *Serapias*, it seems more appropriate to discuss conditionally that it could be affected by climate change according to IPCC scenarios. However, this effect appears more likely in those orchids with narrower bioclimatic ranges across all studied parameters.

**Author Response:**

 Thank you, reviewer for your comments.  We have changed in the text the wording to clarify the ideas in those two conclusions.

**Reviewer Comments:**

- Methods, Data compilation.

Please could you change "Excel data-base" by "Excel file"? It refers to a spreadsheet program rather than a database management system. In the same paragraph, could you also provide the proper reference for *Flora Iberica*? It is listed as number 11 in the bibliography.

 Regarding the WorldClim database, it is well-known and widely used by the scientific community. However, the authors should include the following reference alongside the link, as proposed in the web site: Fick, S.E. and R.J. Hijmans, 2017. WorldClim 2: new 1 km spatial resolution climate surfaces for global land areas. International Journal of Climatology 37 (12): 4302-4315.

**Author Response:**

 Thank you, reviewer, we have modified your comments in the text, please see section 2. Methods in the final version of the manuscript

**Reviewer Comments:**

- Phytosociological adscription and conservation status:

In the second paragraph: ‘…regional, national or international levels’ instead ‘…regional, national or international level’.

**Author Response:**

 We have modified that issue.

**Reviewer Comments:**

- Results and discussion:

Please, review some ‘subsp.’ written in italics in the caption of the Results and discussion section.

**Author Response:**

 Thank you, reviewer, we have modified the format of some subsp. That were in italics in the text.

**Reviewer Comments**

- Phytosociological adscription and conservation status:

In the third paragraph, please review the last sentence; it seems that something is repeated.

**Author Response:**

 Thank for your appraisal, que have modified the repetition

# Reviewer report: Reviewer response for version 1

**DOI:** 10.21956/openreseurope.19174.r45028

**Published:** 2024-10-24

**Authors:** Carlos José Pinto Gomes

**Affiliations:** 1 Environment and Planning, Institute for Mediterranean Agrarian and Environmental Sciences (ICAAM), School of Science and Technology,, University of Évora (Portugal),, Évora, Portugal

This work highlights the importance of utilizing biogeographic units (district level), supported by bioclimatology and phytosociology, in the conservation of the orchids addressed in this study.

 Overall, the Introduction section is well written and informative. However, in the second paragraph, clarification is suggested regarding “reducing tree density.” How does lower tree density contribute to the resilience of forests?

 In the last sentence of the Introduction, why is the phrase in the singular? “This species” should be replaced with “these species.”

 At the end of the Introduction, the authors clearly present the objectives of the study.

 The Methodology is well suited to the objectives defined by the authors.

 The Results and Discussion are presented very clearly and do not raise any doubts.

 This work should be regarded as a reference for future studies that address plants, communities, and habitats of significant community interest.

Is the study design appropriate and does the work have academic merit?

Yes

Is the work clearly and accurately presented and does it cite the current literature?

Yes

If applicable, is the statistical analysis and its interpretation appropriate?

Yes

Are all the source data underlying the results available to ensure full reproducibility?

Partly

Are the conclusions drawn adequately supported by the results?

Yes

Are sufficient details of methods and analysis provided to allow replication by others?

Yes

Reviewer Expertise:

This work highlights the importance of utilizing biogeographic units (district level), supported by bioclimatology and phytosociology, in the conservation of the orchids addressed in this study. Overall, the Introduction section is well written and informative. However, in the second paragraph, clarification is suggested regarding “reducing tree density.” How does lower tree density contribute to the resilience of forests? In the last sentence of the Introduction, why is the phrase in the singular? “This species” should be replaced with “these species.” At the end of the Introduction, the authors clearly present the objectives of the study. The Methodology is well suited to the objectives defined by the authors. The Results and Discussion are presented very clearly and do not raise any doubts. This work should be regarded as a reference for future studies that address plants, communities, and habitats of significant community interest.

I confirm that I have read this submission and believe that I have an appropriate level of expertise to confirm that it is of an acceptable scientific standard.

# Response

**Published:** 2025-01-17

**Authors:** Alejandro González Pérez

**Affiliations:** Biodiversity and Environmental Management (Botany), Universidad de Leon, León, Castile and León, Spain

**Reviewer Expertise -**

 This work highlights the importance of utilizing biogeographic units (district level), supported by bioclimatology and phytosociology, in the conservation of the orchids addressed in this study. Overall, the Introduction section is well written and informative. However, in the second paragraph, clarification is suggested regarding “reducing tree density.” How does lower tree density contribute to the resilience of forests? In the last sentence of the Introduction, why is the phrase in the singular? “This species” should be replaced with “these species.” At the end of the Introduction, the authors clearly present the objectives of the study. The Methodology is well suited to the objectives defined by the authors. The Results and Discussion are presented very clearly and do not raise any doubts. This work should be regarded as a reference for future studies that address plants, communities, and habitats of significant community interest.

**Author Response -**

 Thank you, reviewer, for taking the time to assess this paper. Your insights and suggestions have been extremely helpful. Your expertise and guidance have made a significant difference in the quality of the paper. Besides, your feedback has been invaluable in helping us to improve our writing and the comments to do so. Each of them is answered point by point in blue below.

 The second paragraph, clarification is suggested regarding “reducing tree density.” How does lower tree density contribute to the resilience of forests? The authors agree with you and the sentence has been addressed as “reducing tree density to decrease competition for water and nutrients during dry periods, and introducing resilient species to drought” and we have included two new citations:

 1) Zamora-Pereira, J.C., Yousefpour, R., Cailleret, M. *et al.* Magnitude and timing of density reduction are key for the resilience to severe drought in conifer-broadleaf mixed forests in Central Europe. *Annals of Forest Science* **78**, 68 (2021). https://doi.org/10.1007/s13595-021-01085-w

 2) Cavender-Bares, J., Grossman, J. J., Guzmán Q., J. A., Hobbie, S. E., Kaproth, M. A., Kothari, S., Lapadat, C. N., Montgomery, R. A., & Park, M. (2024). Forest and Biodiversity 2: A tree diversity experiment to understand the consequences of multiple dimensions of diversity and composition for long-term ecosystem function and resilience. *Methods in Ecology and Evolution*, 15, 2400–2414. https://doi.org/10.1111/2041-210X.14435

 - In the last sentence of the Introduction, why is the phrase in the singular? “This species” should be replaced with “these species.”.

 Thank you, reviewer, we have modified that word in the text
